# Supplementary material for: Crystal structure of an RNA-cleaving DNAzyme
Source: Nat Commun. 2017 Dec 8;8:2006. doi: 10.1038/s41467-017-02203-x (PMC5722873; doi:10.1038/s41467-017-02203-x)
Supplement: Supplementary file 1 — Supplementary information [file 41467_2017_2203_MOESM1_ESM.pdf]

Supplementary Figures

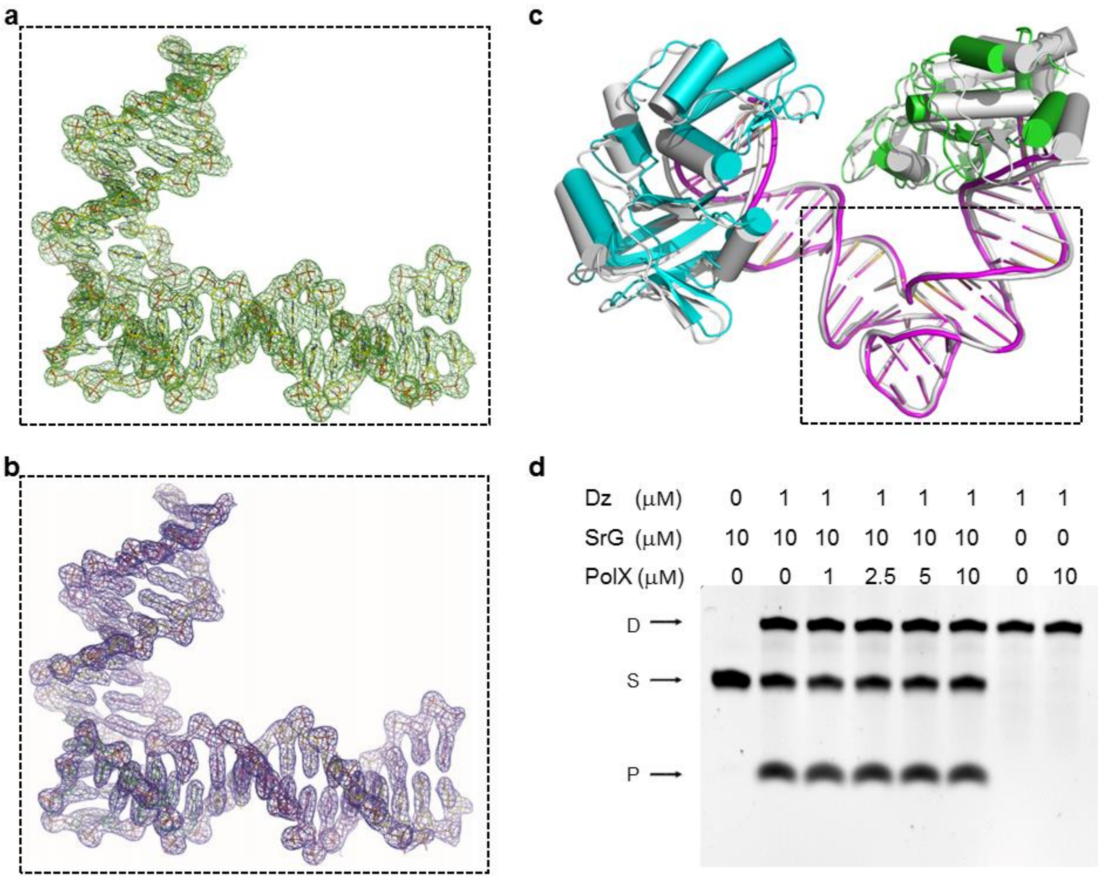

Supplementary Figure 1. Overall fold of 8-17 DNAzyme complex. (**a**) The annealed F_o-F_c omit map (contoured at 3.0 σ level) and (**b**) the refined $2F_o$-F_c map (contoured at 1.5 σ level) of the DNAzyme complex, based on the DNAzyme-Pb^{2+} structure. (**c**) Superposition of the DNAzyme-Pb^{2+} structure and the DNAzyme(2'-OMe-G) structure, based on the catalytic core and the flanking base pairs, which are highlighted with black box. The DNAzyme(2'-OMe-G) structure is colored in white, whereas, the *Asfv*Polx proteins are colored in cyan or green and DZ36 and DNA substrate are colored in magenta in the DNAzyme-Pb^{2+} structure. (**d**) *in vitro* cleavage assays in the presence or absence of *Asfv*PolX. DNAzyme (Dz36), substrate (SrG), and product are indicated by black arrows and labelled as D, S, and P on the gel, respectively.

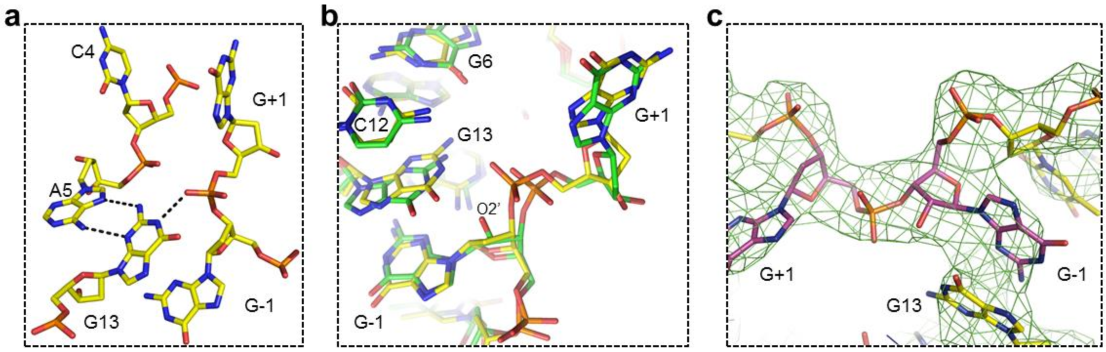

Supplementary Figure 2. Conformational comparison of the catalytic site residues. (**a**) The H-bond interaction between the N1 atom of G13 and the OP1 atom of G+1 observed in the DNAzyme-Pb^{2+} structure. (**b**) Superposition of the DNAzyme-Pb^{2+} and DNAzyme(2'-OMe-G) structures showing the local conformational changes. (**c**) The annealed F_o-F_c omit map (contoured at 3.0 σ level) showing the detailed conformations of G-1 and G+1 residues observed in the DNAzyme(2'-OMe-G) structure. In panel **b**, The C atoms are colored in yellow and green for the DNAzyme-Pb^{2+} structure and the DNAzyme(2'-OMe-G) structure, respectively.

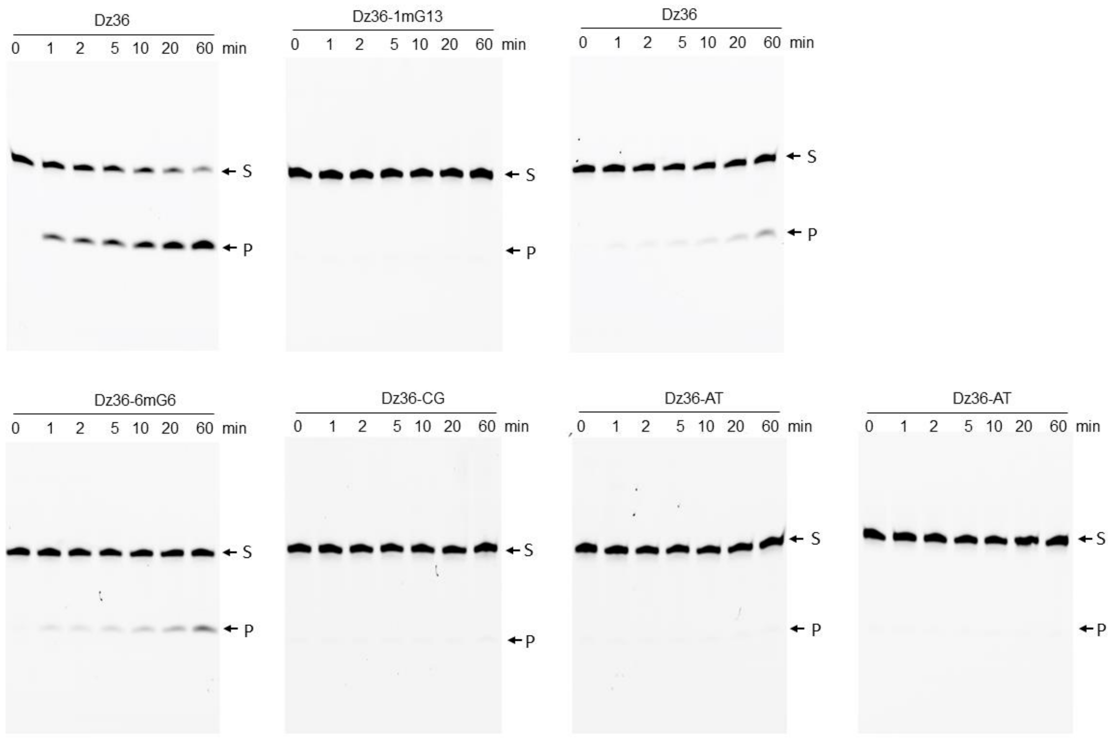

Supplementary Figure 3. *in vitro* cleavage assays of the wild-type DNAzyme (Dz36), mutants with methylated G6 or G13, and mutants with G6:C12 replaced with other Watson-Crick base pairs. *S*ubstrate SrG is utilized in all the reactions. The substrate and product are indicated by black arrows and labelled as S and P on the gel, respectively.

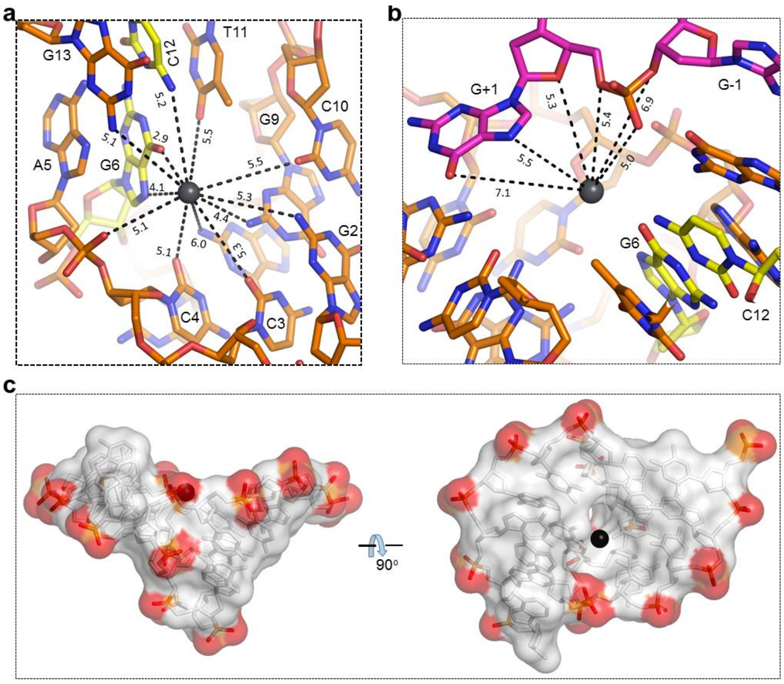

Supplementary Figure 4. The Pb^{2+} binding cage. The detailed distances between Pb^{2+} and the heteroatoms of the surrounding residues of (**a**) the DNAzyme and (**b**) the GG-kink of the substrate. (**c**) Surface representation showing the Pb^{2+}-binding pocket and the distributions of the backbone phosphate groups. The Pb^{2+} ion is shown as black sphere in all panels. In panel **c**, the phosphorus atoms and the oxygen atoms of the backbone phosphate groups are colored in orange and red, respectively.

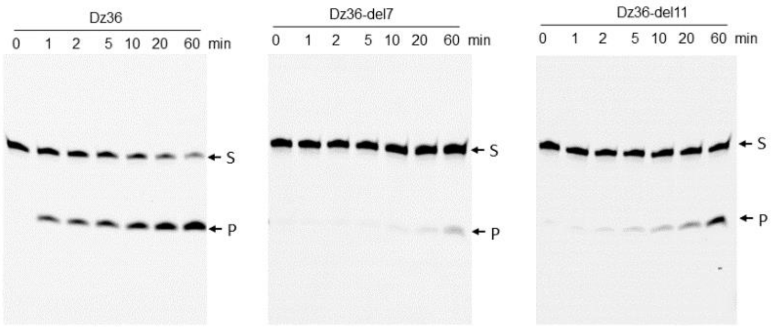

Supplementary Figure 5. *in vitro* cleavage assays of the wild-type DNAzyme (Dz36), mutants with C7 or T11 deletion. Substrate SrG is used in all the reactions. The substrate and product are indicated by black arrows and labelled as S and P on the gel, respectively.

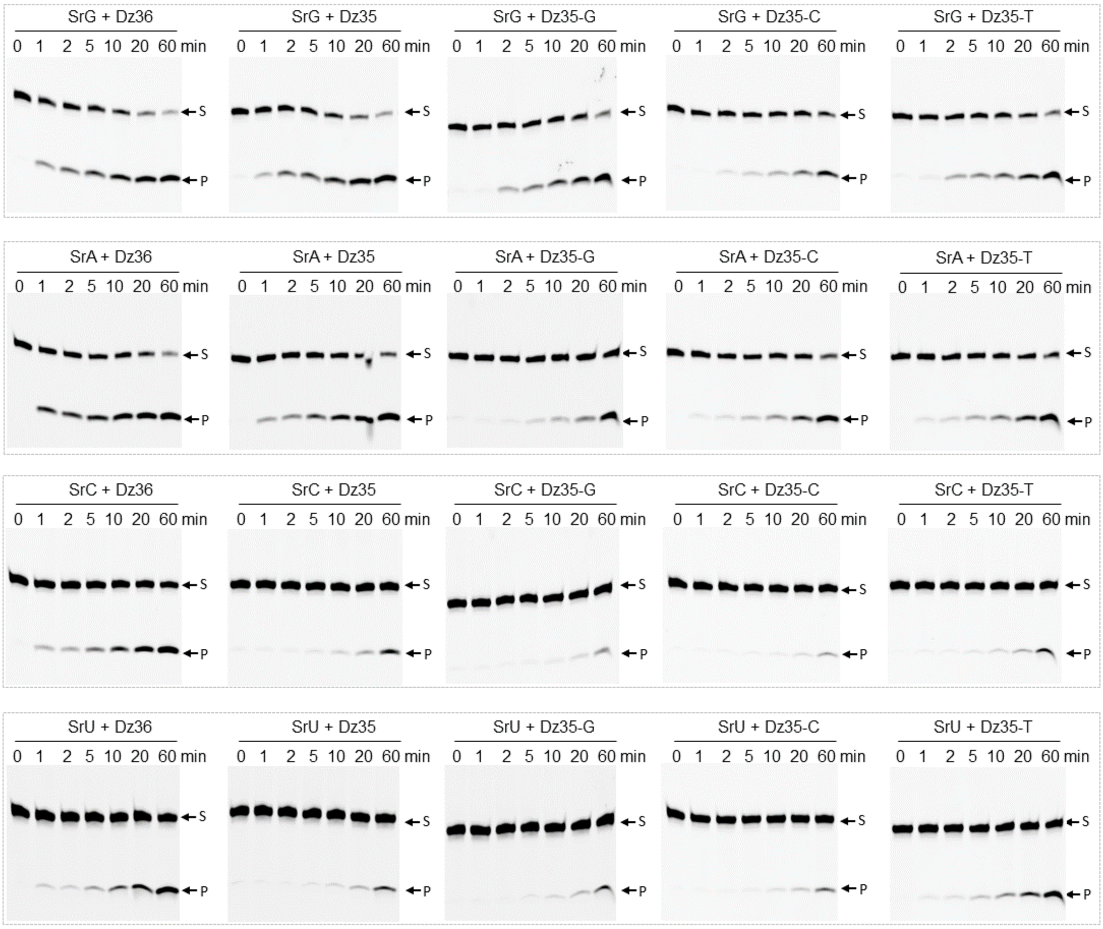

Supplementary Figure 6. *in vitro* cleavage assays of the wild-type DNAzyme (Dz36), mutant with A15 deletion (Dz35), and mutants with both A15 deletion and A14 mutation (Dz35-G, Dz35-C, Dz35-T). DNA substrates with single rG residue (SrG), rA residue (SrA), rC residue (SrC), or rU resiude (SrU) are used in the assays. The substrate and product are indicated by black arrows and labelled as S and P on the gel, respectively.

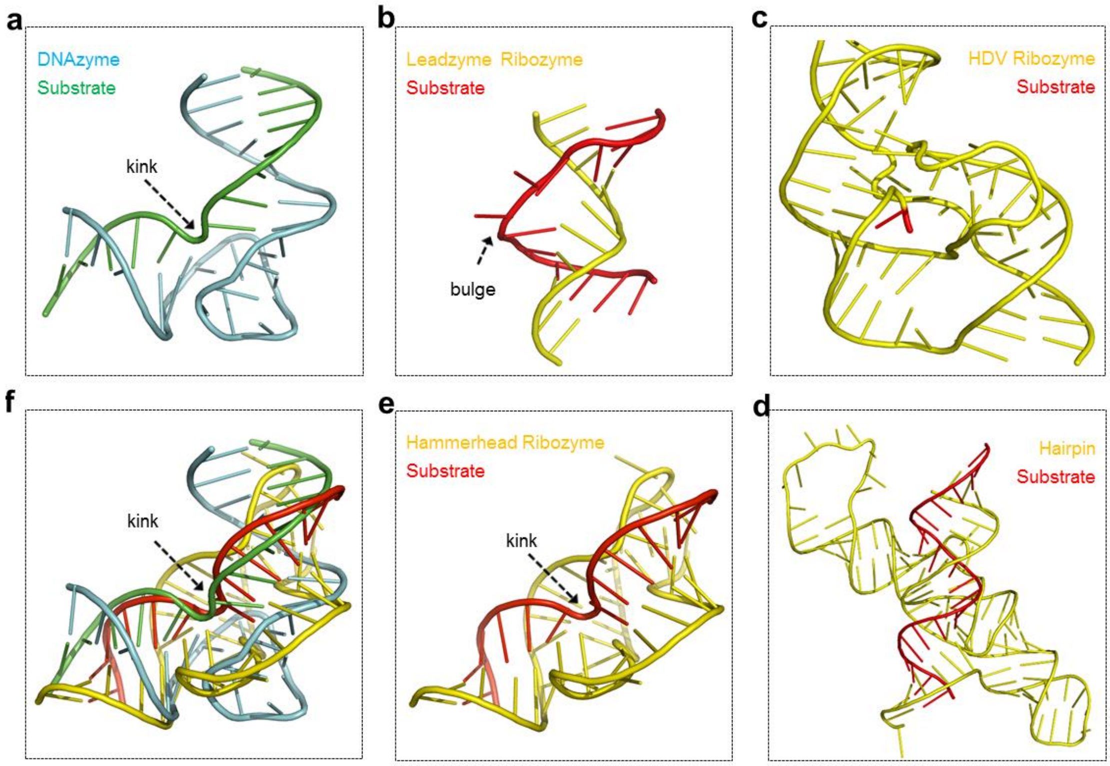

Supplementary Figure 7. Structural comparison between 8-17 DNAzyme and RNA-cleaving ribozymes. Overall structure of (**a**) the DNAzyme complex, (**b**) the leadzyme complex, (**c**) the HDV complex, (**d**) the hairpin complex, and (**e**) the hammerhead complex. (**f**) Superposition of the DNAzyme and the hammerhead ribozyme complexes, based on the substrate strands. For the DNAzyme structure, the enzyme and the substrate are colored in cyan and green, respectively. For all the ribozyme structures, the enzyme and the substrate are colored in yellow and red, respectively.

Supplementary Table 1. Sequence of the DNAzymes and the substrates

Name	Sequence (from 5' to 3')[a]
Numbering	TGTAACGCAC**TGCCAG-CGGC-TCGAA**ATCTCTCTCGT<br>**123456 78910**<br>**11**<br>**12**<br>**13**<br>**14**<br>**15**
Dz36	TGTAACGCAC**TGCCAG-CGGC-TCGAA**ATCTCTCTCGT
Dz36-1mG13	TGTAACGCAC**TGCCAG-CGGC-TC(1mG)AA**ATCTCTCTCGT
Dz36-6mG13	TGTAACGCAC**TGCCAG-CGGC-TC(6mG)AA**ATCTCTCTCGT
Dz36-6mG6	TGTAACGCAC**TGCCA(6mG)-CGGC-TCGAA**ATCTCTCTCGT
Dz36-CG	TGTAACGCAC**TGCCAC-CGGC-TGGAA**ATCTCTCTCGT
Dz36-AT	TGTAACGCAC**TGCCAA-CGGC-TTGAA**ATCTCTCTCGT
Dz36-TA	TGTAACGCAC**TGCCAT-CGGC-TAGAA**ATCTCTCTCGT
Dz36-del7	TGTAACGCAC**TGCCAG--GGC-TCGAA**ATCTCTCTCGT
Dz36-del11	TGTAACGCAC**TGCCAG-CGGC--CGAA**ATCTCTCTCGT
Dz35	TGTAACGCAC**TGCCAG-CGGC-TCGA-**ATCTCTCTCGT
Dz35-G	TGTAACGCAC**TGCCAG-CGGC-TCGG-**ATCTCTCTCGT
Dz35-C	TGTAACGCAC**TGCCAG-CGGC-TCGC-**ATCTCTCTCGT
Dz35-T	TGTAACGCAC**TGCCAG-CGGC-TCGT-**ATCTCTCTCGT
SrG	ACGAGAGAGAT**rGG**GTGCGTTACA
SrA	ACGAGAGAGAT**rAG**GTGCGTTACA
SrC	ACGAGAGAGAT**rCG**GTGCGTTACA
SrU	ACGAGAGAGAT**rUG**GTGCGTTACA
DNA-G	ACGAGAGAGAT**-GG**GTGCGTTACA

[a]Catalytic core and the kink site residues are highlighted in bold.
